# A tumor-microenvironment-activated nanoplatform of modified SnFe_2_O_4_ nanozyme in scaffold for enhanced PTT/PDT tumor therapy

**DOI:** 10.1016/j.heliyon.2023.e18019

**Published:** 2023-07-05

**Authors:** Yang Shuai

**Affiliations:** a College of Life Science and Technology, Huazhong University of Science and Technology. 430074, China

**Keywords:** Antitumor, Phototherapy, SFO nanozyme, Polydopamine, Scaffold

## Abstract

Phototherapy has attracted widespread attention for cancer treatment due to its noninvasiveness and high selectivity. However, severe hypoxia, overexpressed glutathione and high levels of hydrogen peroxide (H_2_O_2_) of tumor microenvironment limit the antitumor efficiency of phototherapy. Herein, inspired by the specific response of nanozymes to the tumor microenvironment, a simple and versatile nanozyme-mediated synergistic dual phototherapy nanoplatform is constructed. In this study, tin ferrite (SnFe_2_O_4_, SFO) nanozyme as a photosensitizer was surface modified with polydopamine (denoted as P-SFO) and incorporated into poly(l-lactide) to fabricate an antitumor scaffold fabricated by selective laser sintering. On one hand, SFO nanozyme could act as a photoabsorber to convert light energy into heat for photothermal therapy (PTT). On the other hand, it played a role of photosensitizer in transferring the photon energy to generate reactive oxygen species (ROS) for photodynamic therapy (PDT). Importantly, its multivalent metal ions redox couples would decompose H_2_O_2_ into O_2_ for enhancing O_2_-dependent PDT and consume glutathione to relieve antioxidant capability of the tumors. Besides, polydopamine as a photothermal conversion agent further enhanced the photothermal performance of SFO. The results revealed the PLLA/P-SFO scaffold possessed a photothermal conversion efficiency of 43.52% for PTT and a high ROS generation capacity of highly toxic ·O_2_^−^ and ·OH for PDT. Consequently, the scaffold displayed a prominent phototherapeutic effect with antitumor rate of 96.3%. In addition, the PLLA/P-SFO scaffolds possessed good biocompatibility for cell growth. These advantages endow PLLA/P-SFO scaffold with extensive applications in biomedical fields and opened up new avenue towards nanozyme-mediated synergistic phototherapy.

## 1 Introduction

Phototherapy has aroused wide attention in tumor treatment due to its spatiotemporal selectivity and low side effect [1]. It mainly includes photothermal therapy (PTT) and photodynamic therapy (PDT). Especially, the combination of PTT and PDT holds great potential for synergistic antitumor therapy [2,3]. PTT uses a photoabsorber to convert photoenergy into heat for tumor ablation and simultaneously PDT utilizes a photosensitizer to transfer the photoenergy to generate reactive oxygen species (ROS) for destroying tumor cells upon laser irradiation [4,5]. It is regrettable that the excessive heat of PTT would cause damage to the surrounding normal cells, the hypoxia condition of tumor microenvironment will deteriorate the O_2_-dependent PDT efficiency and the overexpressed glutathione as antioxidant in tumor microenvironment might weaken the ROS oxidative damage effect [6].

In recent years, developing suitable photosensitizer for tumor therapy has received much interest and advances in nanotechnology have led to the development of nanomaterials based photosensitizers. Nanomaterials with singlet oxygen generation properties, such as aggregation-induced emission (AIE) nanodots, carbon-based quantum dots and ultra-small metal nanoclusters, can overcome most of the limitations of traditional photosensitivities through molecular design and engineering [[7], [8], [9]]. Among them, AIE photosensitizers was widely used in the biomedicine communities owing to their intrinsic fluorescence which could be “turned on” in the aggregate state for disease monitoring while generating singlet oxygen species for PDT treatment [10,11]. For example, Yaraki et al. [12] applied Au585@AIE-PS nanodots for simultaneous fluorescence imaging and photodynamic ablation of HeLa cancer cells with strongly enhanced PDT efficiency in vitro, which provided a better understanding of the metal-enhanced AIE-PS nanohybrid systems and opened up new avenue towards advanced image-guided PDT with greatly improved efficacy.

Nanozymes are a class of nanomaterials with enzymatic catalytic characteristics, which can simulate catalase enzymatic activity to convert H_2_O_2_ into O_2_ to enhance O_2_-dependent PDT or mimic glutathione peroxidase-like activity to consume glutathione in tumor microenvironment [13,14]. It has been reported that a photoresponsive nanocatalytic platform could be designed for enhanced PTT/PDT tumor therapy. For example, Chen et al. [15] developed a composite nanosystem with catalase activities and PTT/PDT synergistic effect by loading Pt nanozyme with a photosensitizer chlorin e6, which exhibited the high photothermal conversion efficiency of 52.6% and showed 80.0% killing rate against HeLa cell under hypoxic conditions. Yang et al. [16] proposed a synergistic therapeutic system with multiple photoactivities and enzymatic catalytic activities by combining iron phthalocyanine nanozyme and hollow nitrogen-doped carbon nanospheres, which possessed the photothermal conversion efficiency of 38.7% and realized a high tumor inhibition rate of 96.2%.

Tin ferrite (SnFe_2_O_4_, SFO) is a photoresponsive nanozyme, which can act as a photoabsorber as well as a photosensitizer to convert light energy into heat for PTT and transfer the photon energy to generate reactive oxygen species (ROS) for PDT. Importantly, its multivalent metal ions (Sn^2+^/Sn^4+^ and Fe^2+^/Fe^3+^) redox couples can exhibit catalase enzymatic activity to decompose endogenous H_2_O_2_ into oxygen (O_2_) and consume GSH to relieve antioxidant capability of the tumors [17,18]. Polydopamine (PDA) as the near-infrared spectrum (NIR) light-responsive materials is capable of absorbing and transferring NIR optical energy into heat [19,20]. It possesses a photothermal conversion efficiency of ∼35%, which has been widely utilized as a photothermal agent for cancer therapy [[21], [22], [23]]. As a consequence, polydopamine is expected to further enhance the photothermal performance of SFO.

Herein, a strategy of utilizing hyperthermia to accelerate catalytic reaction process for nanozymes in scaffold was proposed. In detail, SFO nanozyme was synthesized and surface modified using PDA to improve the photothermal effect, and thereby enhancing the enzymatic catalytic activities for PTT/PDT synergistic therapeutic effect of nanozymes. Afterwards, the modified SFO (P-SFO) was incorporated into poly(l-lactide) (PLLA) to fabricate the 3D scaffolds by selective laser sintering (SLS) for bone cancer [24]. The microstructure and chemical composition of SFO before and after PDA coating were analyzed. The H_2_O_2_ consumption as well as O_2_ production ability of P-SFO were evaluated. The photothermal performance of the scaffold was studied and the ROS generation capacity of was evaluated. The PDT effect of the scaffolds was evaluated and the mechanism of electrons and holes photoexciting from valence band to conduction band was discussed. The synergistic anticancer performance of the scaffolds was tested by confocal laser scanning microscopy, CCK8 assay and flow cytometry.

## 2 Materials and methods

### 2.1 Materials

PLLA powder was provided by Shenzhen Polymtek Biomaterial Co., Ltd, China. SnCl_2_·2H_2_O and FeCl_2_·4H_2_O were obtained from Suzhou Bin Shun Chemical Industry Co., Ltd, China. NaOH and NH_4_OH were provided by Aladdin Co., Ltd, China. Ethylene glycol was purchased from Suzhou Jun He Chemical Material Co., Ltd, China. Dopamine hydrochloride and Tris HCl buffer solution was obtained from Sigma-Aldrich Co., Ltd, China. Anhydrous ethanol Anhydrous ethanol was provided by Chengdu Jinshan Chemical Reagent Co., Ltd. China. Deionized water was collected from the deionized water faucet in the laboratory using a Barnstead Nanopure system.

### 2.2 Synthesis of polydopamine modified SFO nanoparticles

Solvothermal method was adopted to synthesize the SnFe_2_O_4_ (SFO) nanoparticles. Firstly, 2.71 g SnCl_2_·2H_2_O and 4.77 g FeCl_2_·4H_2_O were dissolved into 100 mL ethylene glycol under magnetic stirring for 2 h. And then the mixed solution was adjusted to pH > 10 by NH_4_OH solution and NaOH. It was then shifted into a 150 mL Teflon-lined autoclave and heated to 180 °C for 18 h. After cooling the autoclave to room temperature, the samples were collected by centrifugation with a speed of 5000 rpm for 10 min and washed repeatedly with deionized water for three times. Finally, the collected precipitate was dried at 65 °C for 6 h in an oven to obtain the final product SFO. Polydopamine (PDA) modified SFO powder was prepared through the oxidative self-polymerization of its monomer (dopamine). Briefly, 2 g SFO powder was mixed with 1 g dopamine hydrochloride in 100 mL Tris HCl buffer solution (10 mM, pH = 8.5) under magnetic stirrer for 12 h at 60 °C. Then, the mixture was centrifuged with a speed of 5000 rpm for 10 min and washed with deionized water for three times to remove unreacted dopamine. At last, the modified SFO powder was dried for 12 h at 65 °C in an oven, which was denoted as P-SFO.

### 2.3 Fabrication of scaffold

To fabricate PLLA-based composite scaffold with 5 wt% SFO and P-SFO, the detailed process was given as follows: taking the preparation of PLLA with 5 wt% P-SFO (PLLA/P-SFO) scaffold for example: (a) 9 g PLLA were dispersed with anhydrous ethanol in a beaker for 0.5 h; (b) 1 g P-SFO were dispersed with anhydrous ethanol for 1.0 h; (c) P-SFO and PLLA suspension were mixed, afterwards a magnetic stir was applied to the mixed suspension for 2 h; (d) ultrasound equipment were used to further disperse the mixed suspension for 2 h; (e) PLLA/P-SFO precipitate was obtained by centrifugation and collection; (f) the precipitate was dried at 65 °C for 12 h in an oven. Finally, selective laser sintering (SLS) was used to fabricate the scaffolds using the composite powders.

### 2.4 Characterization methods

X-ray diffractometer (XRD) was applied to analyze the phase composition of SFO and P-SFO powders at 5°/min from 2θ = 20–80°. Fourier Transform Infrared Spectroscopy (FTIR) was used to characterize the functional group of SFO and P-SFO powders over the wavenumber range 4000–500 cm^−1^. The surface morphology and crystalline structure of the powder were characterized by a high-resolution transmission electron microscope (HRTEM), and Energy-dispersive spectroscopy (EDS) mapping was carried out for determining the element distribution. The X-ray photoelectron spectroscopy (XPS) was performed using a mono-chromated Al-ka (1486.6 eV) X-ray source operating at the power of 385 W.

### 2.5 Enzymatic catalytic ability measurement

Titanium sulfate (Ti(SO_4_)_2_) spectrophotometric method was used to study the consumption of H_2_O_2_ according to the principle that orange complex can be formed between H_2_O_2_ and Titanium ion (Ti^4+^) in acidic medium. The detailed process was as follows: Firstly, PLLA/P-SFO (10 mg) was evenly dispersed in H_2_O_2_ (5 mL, 1 × 10^−3^ M) solution under ultrasonic for 0.5 h. And then, Ti(SO_4_)_2_ (24%, 665 μL) was diluted with water (25 mL). PLLA/P-SFO solution and Ti(SO_4_)_2_ solution were mixed and stirred at room temperature, and H_2_SO_4_ (4.165 mL) was added into the mixture to ensure the acidic condition. Finally, to evaluate the consumption of H_2_O_2_, SFO nanoparticles were removed by centrifugation and the absorbance of remaining H_2_O_2_ was tested by UV–vis spectroscopy at absorption spectra of 405 nm. The catalase-like activity of PLLA/P-SFO was monitored with a dissolved oxygen meter to evaluate O_2_ concentration. In detail, PLLA/P-SFO (10 mg) was evenly dispersed in 5 mL PBS buffer with various pH (6.5, and 7.4) before adding H_2_O_2_ (5 mL, 40 mM) solution. Finally, and the concentration of O_2_ solution was monitored and recorded at different time points during 20 min using the dissolved oxygen meter.

The depletion of glutathione (GSH) was investigated by 5,5′-dithiobis-(2-nitrobenzoicacid) (DTNB) as a probe since GSH can react with DTNB to produce 2-nitro-5-mercaptobenzoic acid and glutathione disulfide. The samples of PLLA and PLLA/P-SFO were treated with GSH (25 μL, 1 × 10^−3^ M) and DTNB (5 μL, 10 mg mL^−1^) in PBS for 0, 3, 6, 12, and 24 h. Afterwards, the SFO nanoparticles was removed by centrifugation. At last, UV–vis spectroscopy was utilized for characterizing the absorption spectra and GSH-depletion capability was quantitatively analyzed using spectrophotometer method. Moreover, the generation capacity of hydroxyl radical (·OH) was detected by 3,3,5,5-Tetramethylbenzidine (TMB), which can be oxidized by highly active ·OH. The absorbance spectra from 500 to 800 nm were tested by UV–vis spectroscopy. In the same way, 1,3-diphenylisobenzonfuran (DPBF) was used as a probe to assess the generation of superoxide anion (·O_2_^−^).

### 2.6 In vitro photothermal measurement

The photothermal heating property of PLLA, PLLA/SFO and PLLA/P-SFO scaffolds was assessed under Near Infrared Ray (NIR) at 808 nm (1.3 W cm^−2^). The PLLA, PLLA/SFO and PLLA/P-SFO scaffolds were added into Phosphate Buffer Saline (PBS) solution (1.5 mL) followed by exposed to 808 nm laser and the laser was turned off when the temperature reached stable. Afterwards, the photothermal conversion efficiency of PLLA/P-SFO was calculated using the data during the dispersion solution was naturally cooled without irradiation. For the thermal stability measurement, PLLA/P-SFO dispersion solution was exposed to the laser and then the laser was turned off for four cycles and each cycle lasted for 10 min. During those tests, the temperature changes were recorded by a digital thermometer infrared thermal camera, and infrared thermal camera was applied to observe the thermal images of the solutions.

### 2.7 ROS level assay

The generation of ROS in the MG 63 cells was tested by 2,7-dichlorofluorescin diacetate (DCFH-DA). MG 63 cells were cultured and then the culture medium with PBS, PLLA and PLLA/P-SFO was added to the plates. The group irradiated upon 808 nm laser for 5 min was also incubated as above for comparation. Afterwards, MG 63 cells was stained with 5 μM DCFH-DA, which was co-cultured with MG 63 cells for another 30 min. Afterwards, centrifugation was used to collect the suspended cells, and PBS was used to wash the cells. The fluorescence signal was monitored by confocal laser scanning microscopy (CLSM). Afterwards, the production of ROS level was tested by microplate reader. Furthermore, to qualify the species of ROS, Electron Spin Resonance (ESR) analysis was performed with 5,5-dimethyl-1-pyrroline N-oxide (DMPO) as a spin-trapping reagent. The cells suspension was prepared similar as above and the measurements were carried out on a spectrometer equipped with a laser system.

### 2.8 Cytotoxicity measurement

Human mesenchymal stem cells (hMSCs) were used for biocompatibility tests. A live/dead viability/cytotoxicity kit was carried out to evaluate the cytotoxicity. Briefly, hMSCs were seeded and hMSCs were co-cultured with the scaffolds for 1, 4 and 7 d, respectively. After washing with PBS, hMSCs were incubated for 30 min with Calcein AM and propidium iodide (PI). Finally, a confocal microscope was applied to observe the stained hMSCs. To quantitatively analyze the cell viability, the cells were seeded and cultured by the same way. And then, the optical density was measured using a microplate reader.

### 2.9 Antitumor therapeutic efficacy

MG-63 osteosarcoma cells were used to assess the antitumor therapeutic efficacy of the scaffolds, which were cultured and then treated with leach liquor with PLLA, PLLA/SFO and PLLA/P-SFO scaffolds. For comparation, another group was cultured and treated in the same way before irradiation. After cell culture for 3 days, cells were stained with Calcein AM and PI. Green fluorescence identified the live cells of the stained MG-63 cells, whereas red fluorescence identified the dead cells. And the tumor cell killing rate was assessed by the standard CCK8 assay. Moreover, the flow cytometry analysis for investigating the mechanism of cell death was carried out. In the same way, MG-63 cells were cultured and treated with PLLA, PLLA/SFO and PLLA/P-SFO scaffolds, the apoptosis and necrosis assay were performed with Annexin V-FITC/PI kit.

### 2.10 Statistical analysis

Data were presented as mean ± standard deviation. The statistical comparison was assessed using Student's t-test, where labels *, ** and *** represent P < 0.05, P < 0.01 and P < 0.001, respectively.

## 3 Results

**Fig. 1:**
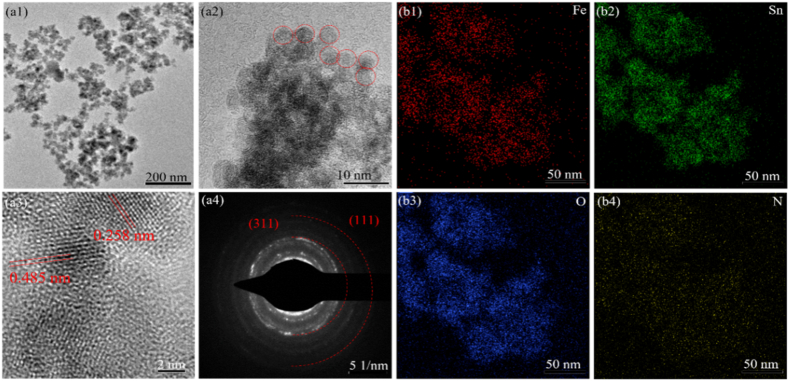
(b1-b2) Transmission electron microscope images of as-synthesized P-SFO at different magnifications. (b3) High resolution Transmission electron microscope image of as-synthesized P-SFO. (b4) Diffraction ring of P-SFO. (c1-c4) Scanning Transmission electron microscope image and elemental mapping of Fe, Sn, O and N of P-SFO.

**Fig. 2:**
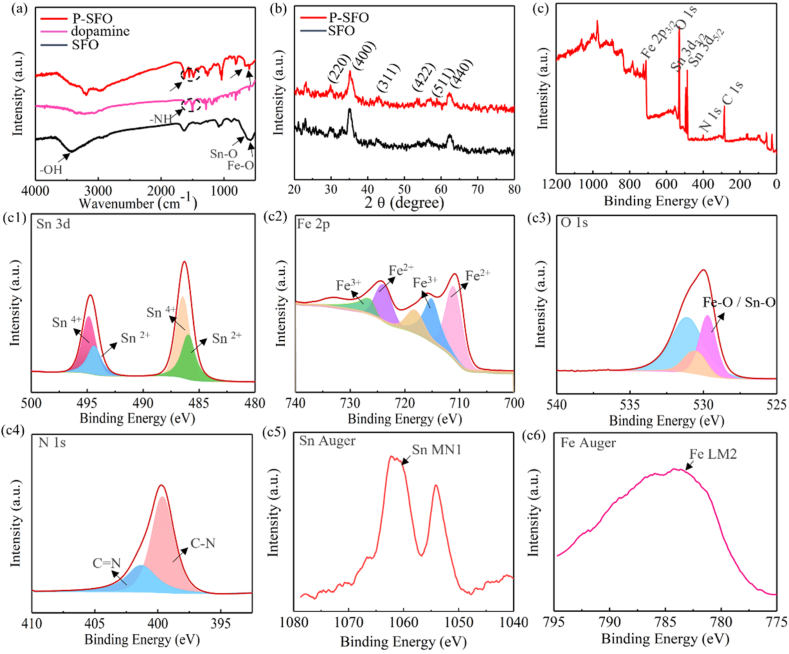
(a) Fourier Transform Infrared Spectroscopy spectra of SFO, dopamine and P-SFO. (b) X-ray diffractometer pattern of SFO and P-SFO. (c) X-ray photoelectron spectroscopy survey spectra. (c1-c4) High-resolution spectra for Sn 3d, Fe 2p, O and N of SFO nanozyme. (c5-c6) The auger spectrum of Sn and Fe.

The morphologies and elementary composition of P-SFO nanozyme were shown in Fig. 1. TEM indicated that P-SFO had a sphere-shaped morphology (Fig. 1(a1, a2)), and the EDS mapping showed a homogeneous distribution of O, Fe, Sn and N (Fig. 1(b1–b4)). In the high-resolution TEM and diffraction ring (Fig. 1(a3, a4)), the lattice fringe shows two characteristic spacing of 0.334 and 0.264 nm, which is corresponding to the (110) and (101) lattice planes of P-SFO, respectively [25]. FT-IR spectroscopy was provided to confirm the functionals groups of PDA, SFO and P-SFO powders. Adsorption peak at 625 cm^−1^ was attributable to the vibration of Sn–O and the band at 584 cm^−1^ corresponding to the vibration band of Fe–O in SFO (Fig. 2(a)) [26]. From the comparison of the two spectra of SFO and P-SFO powders, the band at 1623 and 1465 cm^−1^ refers to N–H stretching vibration of PDA [27], which only existed in P-SFO. The crystalline spinel structure was analyzed by X-ray diffraction (XRD) as shown in Fig. 2(b). According to the results, XRD patterns displayed seven peaks, at 26.6°, 33.9°, 38.0°, 51.8°, 57.8°, 65.9° and 71.3°, corresponding to the (110), (101), (200), (211), (002), (301) and (202) planes, respectively [28].

To further investigate the surface properties of P-SFO nanoparticles, XPS spectra were carried out and the survey spectrum of P-SFO (Fig. 2(c)) indicated O, N, Sn, Fe, and C elements in the samples. Fig. 2(c1) exhibits the high-resolution spectrum of Fe 2p as well as the fitting curves to evaluate the different valence of Fe. The Fe 2p peaks at 709.9 and 715.4 eV belonged to the spin-orbit splitting of Fe 2p3/2 and the other two peaks at 723.4 and 732.3 eV related to the spin-orbit splitting of Fe2p1/2, respectively. Moreover, the peaks at 709.9 eV and 728.6 eV was corresponding to the binding energies of Fe^3+^ and the peaks at 715.4 eV and 732.3 eV was corresponding to the binding energies of Fe^2+^, respectively. Fig. 2(c2) exhibited the high-resolution XPS spectrum of Sn 3d as well as the fitting curves to evaluate the different valence of Sn. The best fitting curves of Sn 3d5/2 with two separate peaks at 485.6 eV and 486.1 eV was corresponding to the binding energies of Sn^2+^ and Sn^4+^, respectively. Similarly, Sn 3d3/2 signal had two peaks at 494.0 and 494.5 eV, which represented the binding energies of Sn^2+^ and Sn^4+^, respectively [29,30].

In addition, it can be found that the auger spectrum of Fe showed a single strong peak locating at 782.8 eV (Fig. 2(c3)) and the auger spectrum of Sn displayed two peaks locating at 1054.7 eV and 1063.2 eV (Fig. 2(c4)), respectively [17]. The high-resolution spectrum of O 1 s spectrum (Fig. 2(c5)) was divided into three main peaks at 529.2, 530.6 and 532.5 eV, which was assigned to the Sn (Fe)–O, lattice oxygen and surface oxygen respectively. The high-resolution spectrum of N 1 s spectrum (Fig. 2(c6)) revealed that the absorption peak at 401.5eV was corresponding to the C–N bond [31].

**Fig. 3:**
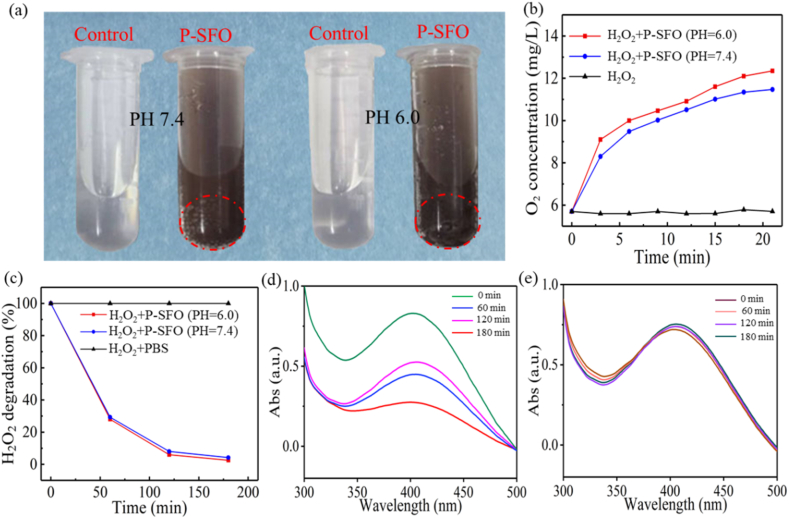
(a) Digital photos for the generation of gas bubbles after the incubation of SFO nanozyme with H_2_O_2_ at different pH values. (b) O_2_ concentration of H_2_O_2_ and SFO nanozyme with H_2_O_2_ at different pH values. (c) H_2_O_2_ degradation of H_2_O_2_ and SFO nanozyme with H_2_O_2_ at different pH values. (d–e) UV–vis spectra of H_2_O_2_–Ti(SO4)_2_ solutions at different times with and without the addition of SFO nanozyme.

It is generally known that the enzymatic activity is affected by pH, and thus, the production of gas bubbles in H_2_O_2_ solution at both pH = 7.4 and pH = 6.0 after adding P-SFO powder was recorded by digital photos (Fig. 3(a)). It could be found that P-SFO powder could produce some O_2_ bubbles after immersing in H_2_O_2_ solution, and the bubbles were more in acidic conditions (pH = 6.0). The concentration of O_2_ was quantitatively analyzed and the result was shown in Fig. 3(b). The concentration of O_2_ in H_2_O_2_ solution without P-SFO powder kept unchanged while in H_2_O_2_ solution with P-SFO powder the concentration of O_2_ continued to rise under both pH values (7.4 and 6.0). The production of O_2_ content almost reached a steady state until 20 min later, which was 11.3 at pH = 7.4 and 12.1 mg/L at pH = 6.0. To further estimate the consumption of H_2_O_2_ quantitatively, we detected the degradation of H_2_O_2_ by time-dependent H_2_O_2_ consumption assay using titanium sulfate (Ti(SO_4_)_2_) as a colorimetric indicator. The degradation ratio of H_2_O_2_ was calculated in Fig. 3(c) and the characteristic absorption peak of H_2_O_2_–Ti(SO_4_)_2_ located at 395 nm was observed in Fig. 3(d and e). The concentration of H_2_O_2_ dropped fastest in the first 20 min, which consumed about 70% of H_2_O_2_. And it took about 200 min for P-SFO to completely consume H_2_O_2_ and in acidic condition H_2_O_2_ was consumed a little more than in neutral condition. The adsorption spectra showed that the peak intensity of H_2_O_2_–Ti(SO_4_)_2_ was gradually decreased for H_2_O_2_ with P-SFO as time prolonged while the peak intensity for H_2_O_2_ with SBF almost kept unchanged, indicating the efficient catalytic activity of P-SFO [32,33].

**Fig. 4:**
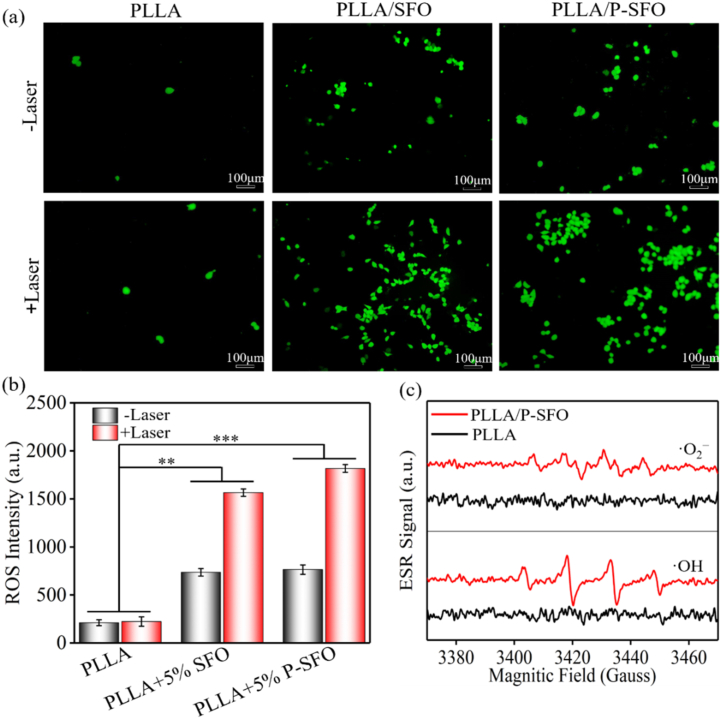
(a) Confocal images of MG-63 cells stained with DCFH-DA to observe reactive oxygen species production of scaffolds with and without laser irradiation. (b) Reactive oxygen species intensity of scaffolds detected by microplate reader. (c) Electron Spin Resonance spectra of ·OH and ·O_2_^−^ trapped by DMPO.

ROS generation was tracked by incubating MG 63 cells with the ^1^O_2_ probe 2′,7′-dichlorofluorescin diacetate (DCFH-DA) with and without NIR laser irradiation (Fig. 4(a)). No distinct green fluorescence was observed in the PLLA group and without laser, indicating no production of the oxidized product DCF. PLLA/SFO group exhibited weak green fluorescence, which could be attributed to the generation of a small amount of ROS by Fenton-like reaction. However, after laser irradiation, the amounts of green fluorescence apparently increased.

A strong green fluorescence was observed in cells treated with PLLA/P-SFO scaffold under NIR laser irradiation, which confirmed the effective intracellular PDT effect of P-SFO caused by hyperthermia under the laser irradiation. And the DCF level by fluorescence spectrophotometers to quantitatively analyze the ROS production (Fig. 4(b)). Consistent with the above confocal laser scanning microscopy results, the group of PLLA/P-SFO under NIR laser irradiation showed the highest fluorescence intensity and the group of PLLA showed the lowest fluorescence intensity. ESR verified the types of ROS using dimethyl-1-pyrroline-N-oxide (DMF) as the trapping agent in Fig. 4(c) [34].

**Fig. 5:**
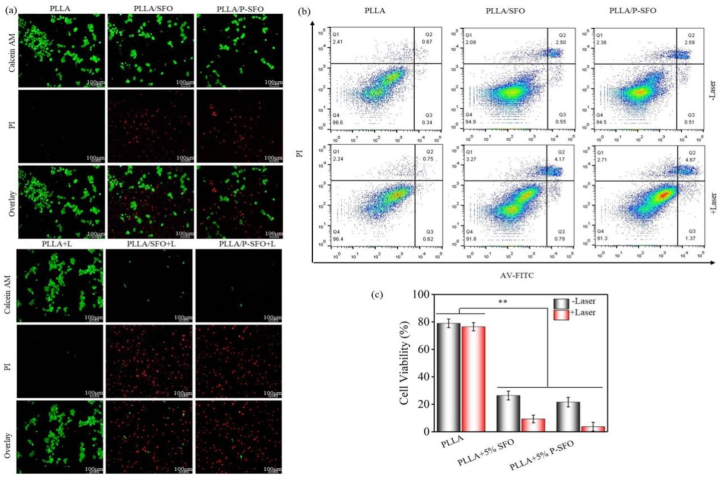
(a) Confocal images of MG-63 cells stained with Calcein AM and PI after incubation with scaffolds for 3 day. (b) Flow cytometry data to show PI-positive dead cells after incubation with scaffolds. (c) Cell viabilities of MG-63 after incubation with scaffolds.

The antitumor effect of scaffolds on MG-63 cells was further assessed via confocal laser scanning microscopy, CCK8 assay and flow cytometry. As shown in Fig. 5(a), the live/dead cell fluorescence staining was observed. From the results, there was almost no dead cells observed for the PLLA scaffolds with and without NIR laser, indicated no tumor killing effect. However, the quantity of the dead cells on PLLA/SFO and PLLA/P-SFO scaffolds increased obviously after the NIR laser irradiation. The quantity of the live and dead cells PLLA/SFO and PLLA/P-SFO scaffolds was quite similar in the without laser irradiation condition. However, as revealed by the strong red fluorescence, more dead cells were observed for PLLA/P-SFO than PLLA/SFO scaffolds, which demonstrated the superior tumor killing efficacy of the PLLA/P-SFO. In addition, CCK8 assay was carried out to quantitively analyze the antitumor rate (Fig. 5(b)). The results revealed that PLLA/P-SFO scaffold possessed the killing efficacy of 78.4% towards MG-63 cells, and the antitumor effect significantly increased upon 808 nm laser irradiation since the cell killing rate of 96.3% was achieved [35]. However, for PLLA scaffold, the MG-63 cell killing efficacy only increased 2.5% with laser compared with that without laser, indicating no PTT/PDT therapeutic potential was observed for PLLA scaffold. Moreover, flow cytometry was carried out and the results were shown in Fig. 5(c). As revealed, a cell survival of 96.6% (without laser irradiation) and 96.4% (with laser irradiation) was obtained for PLLA scaffold, and the percentages of late apoptotic cells were only 0.67% (without laser irradiation) and 0.75% (with laser irradiation) for PLLA scaffold, suggesting slight apoptosis and no antitumor effect. The percentages of late apoptotic cells were detected to be 2.50% and 2.59% for PLLA/SFO than PLLA/P-SFO scaffolds, respectively. The antitumor effect significantly increased and the percentages of late apoptotic cells were increased to 4.17% and 4.67% for PLLA/SFO and PLLA/P-SFO scaffolds under laser, respectively.

**Fig. 6:**
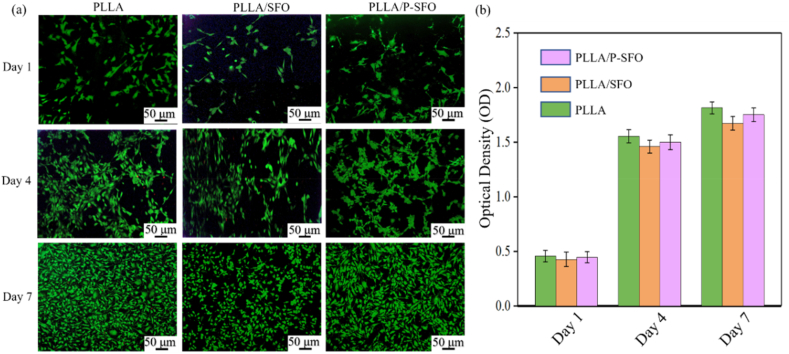
(a) Confocal images of mesenchymal stem cells stained with Calcein AM and PI after incubation with scaffolds for 1, 4 and 7 day. (b) CCK8 assay of the mesenchymal stem cells after incubation with scaffolds for 1, 4 and 7 days.

The cytocompatibility of the scaffolds was evaluated to ensure the biosafety of applying in tissue engineering [36,37]. The fluorescence tests of human mesenchymal stem cells (hMSCs) incubated with the scaffold samples were performed to evaluate the cell toxicity (Fig. 6(a)). The living cells were stained in green, which were cultured for different time periods (1, 4 and 7 days). From the results, the quantity of the living cells increased obviously with the culture time, which implied that the PLLA, PLLA/SFO and PLLA/P-SFO scaffolds were biocompatible to hMSCs. By comparation, the largest number of live cells were observed on the PLLA scaffold. The quantity of the living cells on the PLLA/SFO scaffolds was smaller than that of on the PLLA/P-SFO scaffolds, which showed that the modification of the PDA on SFO improved the cytocompatibility due to the hydrophilic groups of the PDA could regulate the surface of the scaffold to promote cell growth and proliferation [38]. In addition, the CCK-8 assay was carried out to quantitatively analyze the cell proliferation of the PLLA, PLLA/SFO and PLLA/P-SFO scaffolds. The optical density (OD value) was proportional to the number of the living cells. As shown in Fig. 6(b), the OD value exhibited an obvious upward trend as the culture time increased, whereas there was no obvious difference between different scaffolds.

**Fig. 7:**
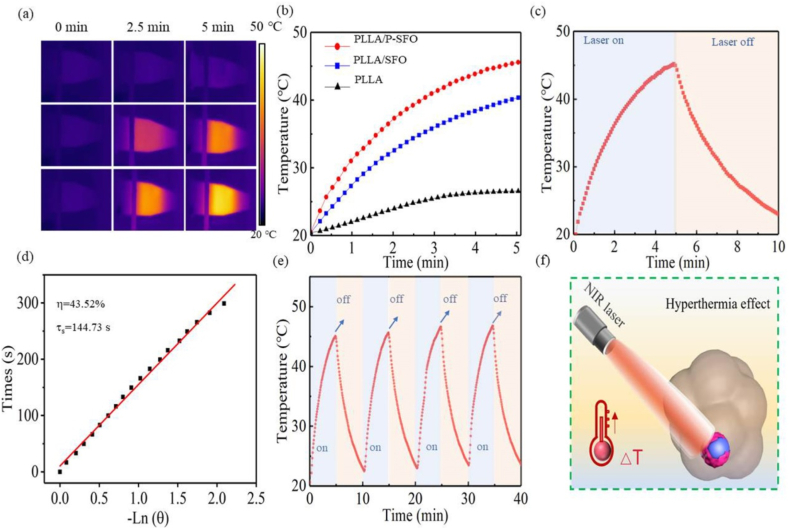
(a) Infrared thermal images of scaffolds. (b) Temperature change curves. (c) Heating and cooling curves. (d) Plot of the cooling time versus the negative natural logarithm of the temperature driving force. (e) Thermal stability of the scaffold (four cycles of laser on/off). (f) Schematic illustration of hyperthermia caused by Near Infrared Ray laser effect.

The photothermal performances of PLLA, PLLA/SFO and PLLA/P-SFO scaffolds were compared by irradiating 808 nm laser as shown in Fig. 7(a–f). Infrared thermography of PLLA, PLLA/SFO and PLLA/P-SFO scaffolds after different irradiation time (0, 2.5 and 5 min) was displayed in Fig. 7(a). It can be found that the PBS solution with PLLA scaffold showed almost no color change while the solution with PLLA/P-SFO scaffold showed a bright yellow after irradiation for 5 min. To further analyze the photothermal performance, the temperature of PBS solution with PLLA, PLLA/SFO and PLLA/P-SFO scaffolds was recorded for irradiation 5 min (Fig. 7(b)). From the results, the temperature increased with the increasing irradiation time. The temperature of PBS solution with PLLA/SFO and PLLA/P-SFO scaffolds had an obvious rise of about 20 °C and 25 °C, respectively. However, the temperature of PBS solution with pure PLLA scaffold had little change, which indicated no hyperthermia effect. PLLA/P-SFO scaffold showed a stronger photothermal performance than PLLA/SFO scaffold since a temperature gap of about 5 °C was observed, resulting from the good photothermal effect of PDA as reported by other studies [[39], [40], [41]]. The heating and cooling profile of PBS solution with PLLA/P-SFO scaffold with maximum temperature changes under irradiation and corresponding cooling time constant (τ) calculation was shown in Fig. 7(c and d), respectively [42]. The photothermal-conversion efficiency (η) of PLLA/P-SFO scaffold at 808 nm was calculated to be 43.52%, which was enough for photothermal therapy. In addition, the photothermal stability of PLLA/P-SFO scaffold was analyzed by recording four recycling temperature changes under 808 nm laser irradiation for 5min (laser on) and subsequently cooled naturally (laser off) as shown in Fig. 7(e). The temperature changes at each cyclic process demonstrated no obvious differences. The hyperthermia effect of PLLA/P-SFO scaffold to kill tumor cells under laser was depicted in Fig. 7(f).

**Fig. 8:**
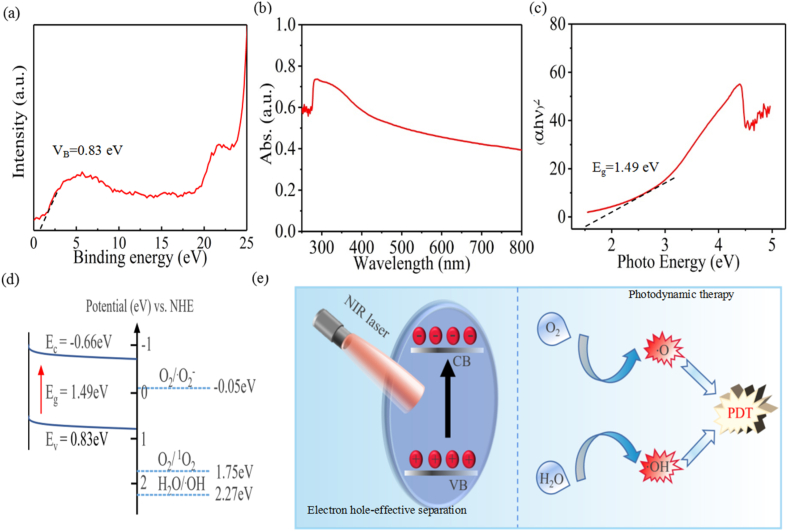
(a) Valence band X-ray photoelectron spectroscopy spectrum of the scaffold. (b) UV–vis diffuse reflectance spectrum of the scaffold. (c) Plots of (αhυ)^2^ and photon energy of the scaffold. (d) Schematic mechanism of reactive oxygen species generation by scaffold. (e) Schematic mechanism of ·OH and ·O_2_^−^ generation by scaffold with laser irradiation.

Excepted from the photothermal therapy effect, photodynamic therapy (PDT) effect was also analyzed Fig. 8(a–e). Based on the fact that a light with photo-energy higher or equal to the bandgap energy of a semiconductor could make electrons and holes photoexcite from the valence band (VB) to the conduction band (CB), and the holes with the same number will remaining in the VB [[43], [44], [45]]. As displayed in Fig. 8(a), the E_VB_ of PLLA/P-SFO was 0.83 eV by XPS VB spectra [46]. The UV–vis diffuse reflectance spectrum of PLLA/P-SFO was displayed in Fig. 8(b). And the Tauc plot showed that the bandgap (E_g_) of PLLA/P-SFO was 1.49 E_V_ (Fig. 8(c)), which was the plots of (αhυ)^1/2^ and photon energy obtained from Fig. 8(b). Correspondingly, according to the equation E_CB_ = E_VB_ – E_g_, the E_CB_ of PLLA/P-SFO is smaller than the E_0_ of O_2_/·O_2_^−^ (−0.05 eV), which gave an energy difference to drive photogenerated e^−^ in CB with O_2_ to generate ·O_2_^−^ (Fig. 8(d and e)).

**Fig. 9:**
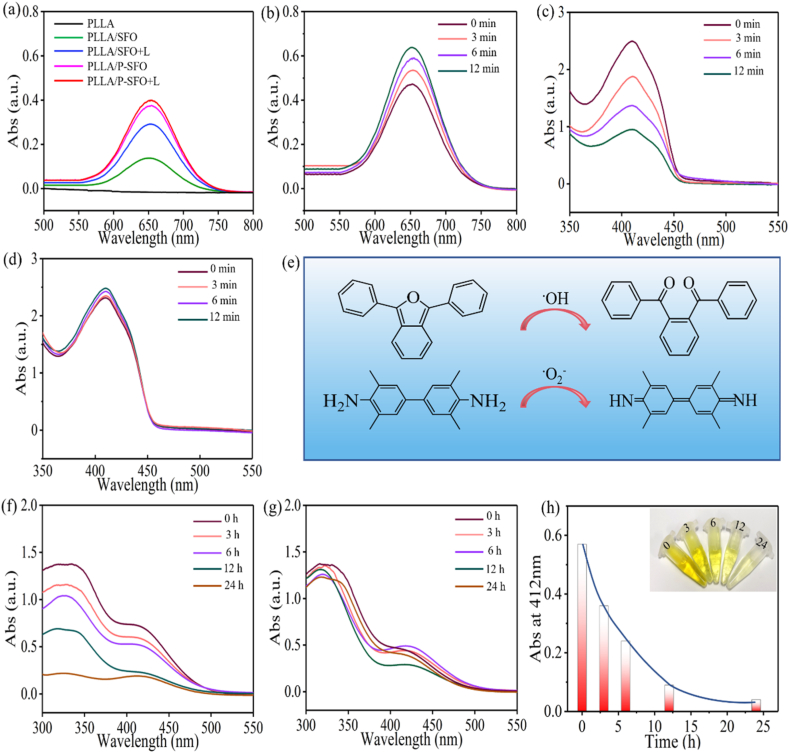
(a) Absorption spectra of TMB to indicate ·OH generation ability for scaffolds, (b) Irradiation time-dependent oxidation of TMB for PLLA/P-SFO scaffold, (c) Irradiation time-dependent oxidation of DPBF to indicate ·O_2_^−^ generation for PLLA/P-SFO scaffold, (d) Irradiation time-dependent oxidation of DPBF to indicate ·O_2_^−^ generation for PLLA scaffold (e) illustration for oxidation process of TMB and DPBF. (f) Absorption spectra of DTNB to indicate GSH depletion for PLLA/P-SFO scaffold. (g) Absorption spectra of DTNB to indicate GSH depletion for PLLA scaffold. (h) Time-dependent GSH depletion for PLLA/P-SFO scaffold.

Considering that Sn^2+^/Sn^4+^ and Fe^2+^/Fe^3+^ redox couple own the Fenton-like reactivity to enhance the intracellular ROS level by consuming H_2_O_2_. The generation capacity of ·OH was detected by using 3,3,5,5-Tetramethylbenzidine (TMB) as probe reagent [47]. The characteristic peak of ·OH at about 652 nm after the incubation with H_2_O_2_ could be found for PLLA/SFO and PLLA/P-SFO, demonstrating the capacity of ·OH production (Fig. 9(a)). Results demonstrated that the peak intensity at 652 nm for both PLLA/SFO and PLLA/P-SFO with laser irradiation was higher than that of no laser. Noteworthy, the peak intensity for PLLA/P-SFO and PLLA/SFO was very close while the intensity of PLLA/P-SFO was prominently higher than that of PLLA/SFO with laser, consisting with the results of Fig. 8 That the modification of PDA improved the photothermal performances of SFO. And the time-dependent ·OH production assay of PLLA/P-SFO under 808 nm laser irradiation was carried out (Fig. 9(b)). Upon increasing the incubation time, the intensity of characteristic peak ·OH was increased significantly.

Furthermore, DPBF was used to detect the production of ·O_2_^−^, which compared the time-dependent ·O_2_^−^ production assay of PLLA/P-SFO and PLLA under laser (Fig. 9(c, d)). The absorption intensity of DPBF gradually lessened upon increasing the laser irradiation time for PLLA/P-SFO. On the contrary, the absorption intensity of DPBF remained unchanged as irradiation time increased for PLLA, indicating the potential of PLLA/P-SFO as promising photosensitizer for PDT by the effective production of ·O_2_^−^ [48,49]. The formula that oxidation of TMB and DPBF through the production of ·OH and ·O_2_^−^ was proposed in Fig. 9(e). It was reported that the highly elevated GSH could neutralize the generated ·OH and ·O_2_^−^, resulting in the undesirable efficacy PDT. Therefore, we further investigated whether P-SFO could mimic GSH oxidase to deplete GSH during therapeutic treatment. The time dependent consumption of GSH for PLLA/P-SFO and PLLA was detected by DTNB indicator. By comparation, the absorption intensity of DTNB gradually lessened as time increased for PLLA/P-SFO while the absorption intensity had no obvious change for PLLA (Fig. 9(f, g)). Moreover, the time dependent GSH-depletion was quantitatively analyzed and the results was presented in Fig. 9(h). It showed that the concentration of GSH decreased sharply for the former 12 h and it was almost consumed completely after 24 h.

## 4 Discussion

FT-IR spectroscopy was provided to confirm the functionals groups of PDA, SFO and P-SFO powders Fig. 2(a). From the comparison of the two spectra of SFO and P-SFO powders, it verified that SFO was modified by the self-polymerization of dopamine successfully. The crystalline spinel structure was analyzed by X-ray diffraction (XRD) as shown in Fig. 2(b). It could be found that the modification did not change the crystalline structure of SFO. The high-resolution spectrum of N 1 s spectrum in (Fig. 2(c6)) revealed that the absorption peak at 401.5eV was corresponding to the C–N bond, which further proved the successful modification of PDA on the surface of SFO. Moreover, the XPS results indicated that both Fe^2+^ and Fe^3+^ are coexisting in p-SFO nanoparticles. Also, it can be concluded that the Sn^2+^ and Sn^4+^ were coexisted in p-SFO nanoparticles. This might be attributed to Fe^2+^ ions were oxidized to Fe^3+^ ions and Sn^2+^ were oxidized to Sn^4+^ by the holes generated in the valence band. The coexistence of Fe^2+^/Fe^3+^ and Sn^2+^/Sn^4+^ redox couples in P-SFO was favorable for Fenton-like, catalase-like, and GSH peroxidase-like activities [50].

The production of gas bubbles in H_2_O_2_ solution revealed that P-SFO nanozyme could decompose H_2_O_2_ to produce O_2_ with the help of Sn^2+^/Sn^4+^ and Fe^2+^/Fe^3+^ as multivalent elements (Fig. 3). And the bubbles were more in acidic conditions than in neutral condition was due to the better catalytic effect. The change of characteristic absorption peak for H_2_O_2_–Ti(SO_4_)_2_ also proved that P-SFO nanozyme was a highly efficient catalyst for consuming H_2_O_2_ to generate O_2_, which was attributed to surmount tumor hypoxia by mass producing O_2_ [51]. Uncontrolled growth of cancer cells can eventually lead to a lack of oxygen in the tumor, which is detrimental to oxygen-based PDT. Tumor tissues will accumulate a large amount of H_2_O_2_, which promotes tumor heterogeneity, neovascularization, invasion and metastasis. Here, P-SFO nanozyme could catalyze the decomposition of overproduced H_2_O_2_ in tumor tissues to enrich oxygen to reinforce the PDT effect, and simultaneously consume H_2_O_2_ to suppress tumor invasion and metastasis.

ROS generation results tested by 2′,7′-dichlorofluorescin diacetate (DCFH-DA) with and without NIR laser irradiation confirmed the effective intracellular PDT effect of P-SFO (Fig. 4(a, b)). The mass production of ROS for PLLA/P-SFO scaffold under laser indicated that the production of O_2_ effectively improved photodynamic effect, which will play a vital role in PDT in hypoxic tumor microenvironment. The reason might be that the hyperthermia effect was contributed to ·OH production since the enhanced temperature could accelerate the ionization process, and therefore increase the Fenton reaction rate [[52], [53], [54]]. Meanwhile, the ESR results also showed that the PLLA/P-SFO scaffold could produce •OH, verified by the four obvious characteristic peaks of ∙OH with an intensity of 1:2:2:1 (Fig. 4(c)). And below Fig. 4(c) Is a typical ESR map of DMPO-∙O_2_, characterized by four peaks with the same peak height and the same distance between adjacent peaks, indicating the production of ∙O_2_. It is known that ROS generated by Fenton-like reaction during PDT played an important role in anti-tumor effect since ROS can kill tumor by destroying their cell membranes, DNA and proteins [[55], [56], [57]].

Encouraged by the excellent ROS generation ability and photothermal effect in vitro, the therapy efficiency of scaffolds on tumor cells was then researched. The results of antitumor tests indicatied that PLLA/P-SFO scaffolds showed combined antitumor efficiency because of photothermal, photodynamic effect and photoenhanced enzymatic catalytic activity to kill tumor cells under laser (Fig. 5). What's more, the percentages of late apoptotic cells were increased for PLLA/SFO and PLLA/P-SFO scaffolds under laser (Fig. 5(b)). Apoptosis can be induced by two major pathways: the mitochondria-mediated pathway (intrinsic) and the death receptor-mediated pathway [58,59]. It might due to an obvious PDT effect by nanozyme-induced O_2_ generation to destroy mitochondria [60]. From the results, PLLA/P-SFO scaffolds possessed great antitumor efficiency due to the nanozyme-mediated synergistic PDT/PTT effect in tumor environment [61]. However, there was no obvious difference between different types of the scaffolds in cytocompatibility experiments as shown in (Fig. 6), indicating the good cytocompatibility of PLLA as a Food and Drug Administration (FDA) approved material for bone tissue engineering [62,63]. Taken together, the results demonstrated no obvious toxicity of the scaffolds to hMSCs and great antitumor efficiency which could be utilized in human tumor therapy [64,65].

The photothermal performances of PLLA, PLLA/SFO and PLLA/P-SFO scaffolds by irradiating 808 nm laser suggested that PLLA/P-SFO scaffold possess good photothermal stability and photothermal-conversion efficiency for PTT (Fig. 7). PTT utilize a photothermal conversion agent that absorbs light energy and converts it into heat energy, resulting in high temperature at the tumor site, destruction of the integrity of cancer cell membrane, and changes in cellular permeability and fluidity that lead to the failure of cell function. At the same time, the protein of cancer cells is denatured, thus inhibiting the replication and expression of cancer cell DNA and inducing the apoptosis of cancer cells. Excepted from the photothermal therapy effect, the photodynamic therapy (PDT) effect of PLLA/P-SFO to generate ROS was also analyzed (Fig. 8). As depicted in Fig. 8(e), the separation efficiency of electron-hole pair could be separated due to the unique nanostructure of the P-SFO and the electron-hole recombination could be prevented by P-SFO as an electron trap. As a result, the excited electrons transferred from the CB instead of recombining with the hole in the VB, which could further react with O_2_ to produce a large amount of ROS (·O_2_^−^ and ·OH) when exposed to the laser irradiation [66].

**Fig. 10:**
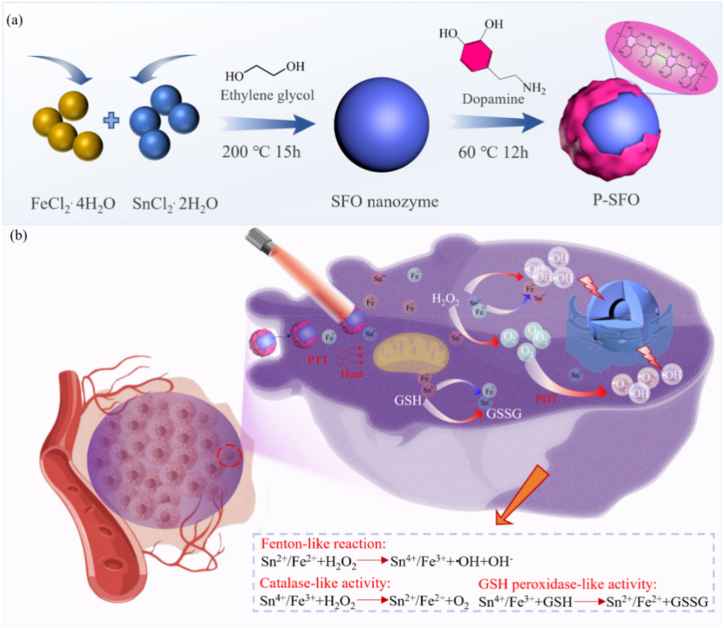
(a) Schematic illustration for the synthetic process of SFO nanozyme modified by polydopamine (P-SFO), by Figdraw. (b) The scheme of therapeutic mechanism for nanozyme-guided synergistic photothermal therapy and photodynamic therapy.

In Fig. 9 the absorption intensity of DPBF indicated the potential of PLLA/P-SFO as promising photosensitizer for PDT by the effective production of ·O_2_^−^. The intensity of characteristic peak ·OH was increased significantly upon increasing the incubation time because of the multivalent Sn^2+^/Sn^4+^ and Fe^2+^/Fe^3+^ redox couple, indicating the catalase-like property of PLLA/P-SFO to reduce H_2_O_2_ into ·OH. It was reported that the highly elevated GSH could neutralize the generated ·OH and ·O_2_^−^, resulting in the undesirable efficacy PDT. Therefore the consumption of GSH was increased as irradiation time with the help of PLLA/P-SFO, since GSH could be oxidized into glutathione disulfide GSSG to remain a high level of ROS for PDT process, and thereby achieving efficient tumor therapeutic activity [[67], [68], [69]]. In conclusion, these results verified the effect of PLLA/P-SFO in producing ROS and consuming GSH for ensuring the therapeutic efficiency of PDT in killing tumor cells. The schematic to depict synthesizing SFO by a hydrothermal method and then modifying SFO by polydopamine (P-SFO) was displayed in Fig. 10(a). And the therapeutic mechanism of P-SFO for cancer therapy with dual phototherapy PTT/PDT was depicted in Fig. 10(b). It can be seen that SFO nanozyme was surface modified with polydopamine, which could produce oxygen and ROS while consuming glutathione and high levels of hydrogen peroxide by Fenton like reaction, Catalase-like and GSH peroxidase-like activity.

## 5 Conclusions

In summary, SFO nanozyme was synthesized and surface modified by PDA to further enhance the photothermal performance. Afterwards, the modified SFO was incorporated into PLLA to fabricate the scaffolds by selective laser sintering for enhanced PDT/PTT tumor therapy. The results revealed that the P-SFO nanozyme could decompose H_2_O_2_ to produce O_2_ for relief of tumor hypoxia and enhancement of the PDT efficiency. In addition, the P-SFO nanozyme also possessed the glutathione peroxidase-like activity, which can consume endogenous glutathione to convert glutathione into its oxidized form. Moreover, the PLLA/P-SFO scaffolds possessed photothermal conversion efficiency of 43.52% for NIR-responsive PTT and a high ROS generation capacity of highly toxic ·O_2_^−^ and ·OH. Thus, the PLLA/P-SFO scaffolds displayed a high antitumor rate of 96.3%, which opened a new horizon for exploring a more powerful tumor treatment nanoplatform.

## Author contribution statement

Yang Shuai: Conceived and designed the experiments; Performed the experiments; Analyzed and interpreted the data; Contributed reagents, materials, analysis tools or data; Wrote the paper.

## Data availability statement

The data that has been used is confidential.

## Declaration of competing interest

The authors declare that they have no known competing financial interests or personal relationships that could have appeared to influence the work reported in this paper.
